# Population‐Based Norms for the Montreal Cognitive Assessment in Arab Adults

**DOI:** 10.1002/brb3.70287

**Published:** 2025-02-09

**Authors:** Iman Amro, Aisha M. Al Hamadi, Alaa A. El Salem, Tawanda Chivese, Stacy S. Wilkins, Salma M. Khaled

**Affiliations:** ^1^ Social and Economic Survey Research Institute (SESRI) Qatar University Doha Qatar; ^2^ Department of Population Medicine College of Medicine Qatar University Doha Qatar; ^3^ Clinical Medicine, David Geffen School of Medicine UCLA Los Angeles California USA

**Keywords:** attention, cognitive function, executive functions, fluency (verbal/nonverbal), Montreal Cognitive Assessment (MoCA), norms/normative studies

## Abstract

**Objective:**

The Montreal Cognitive Assessment (MoCA) is a brief screening instrument for detecting mild cognitive dysfunction, a precursor to many cognitive disorders, such as dementia, which have increased in prevalence globally. Qatar, a small high‐income country, has the largest projected increase in dementia of any country in the Middle East. Yet no population‐based norms for cognitive function are available to date.

**Methods:**

As part of the first national cross‐sectional study of mental health, a total of 395 Qatari and non‐Qatari Arabs, 18–74 years of age, were evaluated face‐to‐face using the Arabic version of the original MoCA (version 7.1). We computed raw and demographically (gender, age in years, and four education categories) adjusted scores for the overall MoCA test and six domains (visuospatial, executive function, attention, language, delayed memory, and orientation). The percentile ranking of raw and adjusted normative (*z*) scores was computed. The 5th percentile ranking was used to derive potential cut‐offs for the overall test and the six related domains.

**Results:**

Female gender, older age, and lower levels of education were associated with poorer overall test scores. The following MoCA overall test and domains cut‐off scores (rounded to the nearest integer) were identified: MoCA (22), visuospatial (2), executive (2.5), attention (4), language (4), and delayed memory (3).

**Conclusions:**

On the basis of our population‐based data, scores below these 5th percentile cut‐offs may warrant further testing and clinical follow‐up for mild cognitive impairment (MCI) in otherwise healthy Arab adults.

## Graphical abstract

In Qatar, being a female, 50 years or older, and with less than 12 years of education were associated with poorer overall test scores on the Montreal Cognitive Assessment (MoCA). Scores below the 5th percentile cut‐offs may warrant further testing and clinical follow‐up for MCI in healthy Arab adults.

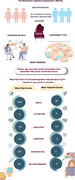

## 1 Introduction

The prevalence of neurocognitive disorders such as Alzheimer's disease and dementia is increasing globally and projected to triple by 2050 (Nichols et al. 2022; Prince et al. 2013). Yet, there are no known cures or established interventions for slowing down the progression of these disorders (Arvanitakis, Shah, and Bennett 2019). Although aging is the strongest known risk factor (van der Flier 2005), mild cognitive impairment (MCI) is an important risk factor for many of these disorders (Nichols et al. 2022). Due to advances in standards of living and healthcare, the proportion of people at risk of cognitive dysfunction, including MCI, is increasing globally, especially in low‐to‐middle‐income countries, or LMICs (Prince et al. 2013) where it is projected that 80% of older people will be living in these countries by 2050 (WHO 2022).

The Montreal Cognitive Assessment (MoCA) is a brief screening instrument used in clinical settings worldwide to detect MCI (Nasreddine et al. 2005) and has several advantages over standard screening tools like the Mini‐Mental Status Examination (Folstein, Folstein, and McHugh 1975). Available in a variety of languages, including Arabic (Nasreddine et al. 2005), the MoCA has also been used as a research tool for measuring the general cognitive status of the population (Fujiwara et al. 2010; Lee et al. 2008; Luis, Keegan, and Mullan 2009; Rossetti et al. 2011).

The MoCA is a short test that assesses six cognitive domains, including visuospatial abilities, executive function, attention, language, delayed recall, and orientation. A value below 26 out of 30 points was validated as a cut‐off score for screening those with MCI (Nasreddine et al.). However, several studies with healthy population samples suggested that country‐specific adjusted norms should be used instead (Poptsi et al. 2019). For example, a Swedish study conducted among 758 healthy showed that 37.3% of their sample (Borland et al. 2017) would be wrongly classified as cognitively impaired using the suggested cut‐off score of 26 derived from the original validation study of the MoCA (Nasreddine et al. 2005).

Demographic variables are important correlates of cognitive performance. Older age is among the most consistently associated with poor performance on cognitive tests, including the MoCA (Bravo and Hébert 1997; Han et al. 2008; Langa et al. 2017; Matallana et al. 2011; Mathuranath et al. 2007; Moraes et al. 2010; Rossetti et al. 2011). Several studies have also shown that lower education is associated with lower MoCA scores in different settings (Conti et al. 2015; Kopecek et al. 2017; Larouche et al. 2016). However, findings in relation to the role of gender are mixed, with some studies suggesting that gender accounts for substantial variance in cognitive screening results on tests like the MoCA (Han et al. 2008; Measso et al. 1993; Mías et al. 2007; Ribeiro et al. 2010; Scazufca et al. 2009), whereas other studies did not support this finding (Bertolucci et al. 2001; Lieberman et al. 1999; Mathuranath et al. 2007; Morgado et al. 2010).

According to the 2019 global burden of disease (GBD) study, countries in the Middle East and North Africa (MENA) are projected to have the largest percentage increases (367%) in the number of dementia cases by 2050 (Nichols et al. 2022). Yet, few studies to date were conducted to screen for MCI or other forms of cognitive impairment in representative samples of the general population in these countries (Hayek et al. 2020; Manee et al. 2017; Muayqil et al. 2021; Rahman and El Gaafary 2009).

Qatar is a small, high‐income country in the MENA region, which has a projected increase in the number of cases of dementia of 1926%—the largest of any country in the Middle East (Nichols et al. 2022). To our knowledge, norms on the MoCA in Qatar have not yet been established. Therefore, the present study aims to determine the raw and sociodemographic‐adjusted normative scores of the overall MoCA test and its domains in a representative sample of Qatar's Arab adult population.

## 2 Methods

### 2.1 Study Design and Population

This study is part of a larger national population study of mental health in Qatar. Details about the larger study were published elsewhere (Khaled et al. 2021). Briefly, the target population was a representative sample of household‐based Qatari and non‐Qatari Arabs, 18 years of age or older, who resided in Qatar at the time of the interview (Khaled et al. 2021). A total of 395 face‐to‐face interviews were completed between January 16 and February 9, 2020 with an overall response rate of 56.9% (Khaled et al. 2021).

### 2.2 Initial Inclusion/Exclusion Procedures

One household occupant was randomly selected for participation in the study. For households with multiple occupants, one potential participant was randomly selected for inclusion in the study from a roster of multiple household occupants. If the selected participant either self‐reported or was reported by proxy (typically the head of the household) to have any overt mental or physical symptoms that prevented them from participating and/or consenting, this participant was excluded, and reasons for exclusion were documented. Physical disabilities, such as poor hearing or vision, poor hand–eye coordination, or compromised mental capacity due to dementia or other degenerative neurological conditions, traumatic brain injury, or severe psychiatric conditions (e.g., schizophrenia), as diagnosed by a health professional, were considered valid reasons for exclusion.

### 2.3 Study Material and Procedures

The MoCA was previously translated to Arabic, back‐translated to English, and cross‐culturally validated, with acceptable psychometric results (Abou‐Mrad et al. 2017). Participants were evaluated using the Arabic version of the original MoCA (version 7.1), available from www.mocatest.org, which was programmed in Blaise 5.2 (Blaise 2017) as part of the larger national study (Khaled et al. 2021).

A total of 29 potential interviewers were trained in how to administer the programmed version of the Arabic MoCA Appendix 1) during the study's face‐to‐face interviews conducted in participants’ households (Khaled et al. 2021). The training sessions spanned over 5 days and included theory and hands‐on practice with the administration of the MoCA module deployed on the interviewers’ laptops. In addition to the training workshop, each interviewer completed at least three supervised scripted role‐play interviews with the MoCA. The trainers scored and evaluated all 29 interviewers, and 27 of them successfully passed the evaluation (Khaled et al. 2021).

Interviewers were evaluated by two (a senior and junior) researchers from the study team. The senior researcher (S.K.) was registered and certified to administer the MoCA, who in turn trained the junior researcher (A.A.) to administer and score the MoCA. The two researchers then trained the laymen interviewers to administer the MoCA as part of the study's interview. Senior neuropsychologist (S.W.), who was also part of the team, supervised both researchers.

After data collection, two researchers (I.A. and A.A.) met with the senior researcher (S.W.) and scored several cases together as a team. Each researcher (I.A. and A.A.) then independently scored all cases thereafter.

## 3 Measures

The MoCA items were segregated into six main domains (visuospatial, executive function, attention, language, delayed memory, and orientation). The attention domain also included concentration and working memory.

The number of years of education was categorized on the basis of the education system in Qatar and the Middle East, with 0–11 years considered less than secondary school, 12 years considered secondary school, 13–15 years considered some post‐diploma education, and 16 years or more considered to have higher post‐secondary education.

### 3.1 Ethics and Informed Consent

The study was carried out according to the principles of the Declaration of Helsinki and received ethical approval from Qatar University's institutional review board (QU‐IRB 1086‐E/19). All individual identifiers and anonymous data were stored in a password‐protected folder on a highly secure university server, which was only accessible by the research team.

### 3.2 Statistical Analyses

All analyses were conducted in STATA version 18 (STATA 2018).

Categorical data were summarized using percentages and frequencies. For MoCA total test score and domain‐based total scores, we presented descriptive statistics, including mean, median, standard deviation (SD), and minimum, maximum, and interquartile range (IQR) values.

For the MoCA test, we calculated the overall test score for each participant by taking the arithmetic sum of all MoCA domains scored as per standard (clinical) scoring guidelines for the test (MoCA 2010). Corresponding relative norms were then estimated on the basis of the entire sample by identifying MoCA test and domain scores that were considered relatively low among our sample of the general (healthy) population, that is, falling within the bottom 5th, 10th, 15th, and 20th percentiles of the sample distribution as per the consensus of the American Academy of Clinical Neuropsychology, which advocates the use of percentiles instead of standard scores for highly restricted score ranges or highly skewed distributions (Guilmette et al. 2020).

We calculated two types of scores for the MoCA test and its domains: raw and adjusted. For the raw scores, we identified the observed test values at or below which the 5th, 10th, 15th, and 20th percentiles of our normative MoCA overall test score and in each of the MoCA domains. Stratified raw mean scores of MoCA overall test by categories of age and education were computed. We also identified expected test values at each of these percentiles by computing the corresponding *z*‐scores based on the mean and the SD from our sample using the following equation, where

(1) $$z=\frac{x-\mu }{\sigma }$$

We also generated demographically adjusted *z*‐scores that can be used in studies and by clinicians to predict a participant score given their demographic characteristics (Abou‐Mrad et al. 2017). This scoring method entailed the following two stages (A and B):

- Linear regression of MoCA score and demographic predictors.

We fitted seven separate linear regression models to the data estimating associations between the same set of sociodemographic variables (gender, age in years, and four education categories) and each of the following dependent variables: MoCA overall test score and six main cognitive domains. The *R*‐squared ($R^{2}$) and root mean squared errors (RMSEs) were reported for all models. Assumptions of linear regression were assessed for the main model with the MoCA overall test score as the dependent variable. Using the “predict” function, adjusted scores were derived from the linear regression models for each participant. This predicted score reflects an estimate of what the participant's score would be given their specific demographic characteristics using the constant and beta coefficients $\hat{(\beta })$.

- Percentile ranking of participants relative to the normative sample.

Next, *z*‐scores were computed by dividing the difference between the predicted and the observed raw scores by the standard error of predictions from the regression line using the following equation:

(2) $$z=\frac{x-\hat{x}}{\sigma }$$

The calculated *z*‐score was then used to determine a participant's percentile ranking relative to the normative sample using the *z*‐distribution. Although we report 5th, 10th, and 15th percentiles of raw and *z*‐scores in this article, we used the 5th percentile ranking to determine “below average” cut‐offs for the overall MoCA score and domains’ scores (Guilmette et al. 2020).

Inter‐rater reliability was computed for two components of the MoCA test (the visuospatial and verbal fluency domains) where two independent raters were involved in the scoring of these domains. For this purpose, we calculated the average intra‐class correlation (ICC) coefficients for consistency using a two‐way mixed model in STATA (https://www.stata.com/manuals/ricc.pdf). The average ICC estimated the agreement between the averages of ratings over the two raters for each of these two domains.

Missing data constituted less than 10% of our data, and those who were missing were assumed to be missing at random for the purpose of our analysis.

## 4 Results

**FIGURE 1:**
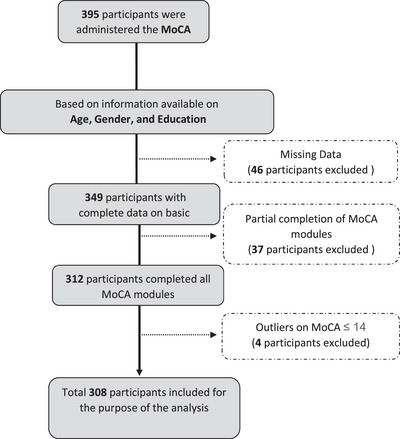
Sample exclusions. MoCA, Montreal Cognitive Assessment.

Of the 395 who participated in the study, a total of 308 records were considered viable for the purpose of our analysis. Subsequent to the data cleaning, 87 participants were excluded from the analysis for various reasons (Figure 1), including missing demographic details (*n* = 46), partial completion of the MoCA (*n* = 37), and outlier MoCA scores (*n* = 4). The mean age of participants was 38 years (SD = 10.2). The mean years of education were 14.2 years (SD = 4.7). Further details about characteristics of the sample are presented in Appendix 2.

The mean, median, and SD for all six MoCA domains are shown in Table 1. The mean for the MoCA overall test score was 23.6 (SD = 3.0), and the median was 24 (IQR = 22–26). Not shown in Table 1, the percentage of participants who scored below the standard clinical cut‐off for the MoCA test score (below a score of 26) was 74.3% (95% CI: 69.2–78.9).

**TABLE 1:** Descriptive statistics for Montreal Cognitive Assessment (MoCA)‐related domain scores, overall score, and main sociodemographic variables.

Domain	Variable	*N*	Mean	SD	Min	Max	Percentile (25%)	Percentile (50%)	Percentile (75%)
**MoCA total**	MoCA‐total	308	23.6	3.0	14.5	29.5	21.5	24	26
**Visuospatial**	Cube drawing	308	0.5	0.5	0	1	0	0.5	1
	Clock drawing	308	1.9	0.8	0	3	1	2	2.5
	Visuospatial total	308	2.4	1.0	0	4	1.8	2.5	3
**Executive function**	Alterating trail making	308	0.5	0.5	0	1	0	0.5	1
	Verbal fluency^a^	308	0.3	0.5	0	1	0	0	1
	Abstraction	308	1.7	0.6	0	2	1	2	2
	Executive function total	308	3.3	1.0	0	5	3	3	4
**Attention**	Digital span forward	308	0.7	0.4	0	1	0	1	1
	Digital span backward^b^	308	0.8	0.4	0	1	1	1	1
	Tapping	308	0.9	0.3	0	1	1	1	1
	Serial subtraction	308	2.6	0.7	0	3	2	3	3
	Attention total	308	5.0	1.1	1	6	4	5	6
**Language**	Naming	308	2.9	0.4	1	3	3	3	3
	Repeating sentence	308	1.4	0.7	0	2	1	2	2
	Language total	308	4.6	1.0	1	6	4	5	5
**Memory—Delayed recall**	Delayed recall total	308	3.5	1.4	0	5	3	4	5
**Orientation index**	Orientation total	308	5.7	0.5	3	6	5	6	6

Abbreviation: SD, standard deviation.

^a^ Counted as part of language in addition to executive function.

^b^ Counted as part of executive function in addition to attention.

The average overall ICC for the visuospatial domain was 0.92 with a 95% CI (0.90, 0.94), whereas the average ICC for the verbal fluency domain was 0.98 (95% CI: 0.98, 0.99).

The mean overall MoCA test scores and corresponding 95% CI varied by age categories from 23.6 (22.7–24.5) in 18–29 years, 23.8 (23.4–24.3) in 30–40 years, and 22.7 (21.4–23.9) in 50+ years.

Among participants in the age category 18–29, the mean scores for the MoCA increased with education years from 22.4 (SD = 3.7) for participants with 0–11 years of education to 24 (SD = 3.2) for those with 16 or more years of education (Table 2). We also observed a similar trend for participants in age categories 30–49 and 50 years or more (Table 2).

**TABLE 2:** Raw mean Montreal Cognitive Assessment (MoCA) scores and corresponding standard deviations (SD) by different age and education categories.

Age categories (Years)	Education level (Years)							
	0–11		12		13–15		16+	
	Mean	SD	Mean	SD	Mean	SD	Mean	SD
**18–29**	22.4	3.7	24.1	2.6	24.2	2.0	24.0	3.2
**30–49**	22.4	3.3	23.4	2.6	23.6	2.7	24.1	2.8
**50+**	19.1	2.5	21.6	3.0	24.3	1.2	23.6	3.1

Among participants with less than 12 years of education, the mean scores for the MoCA decreased with age from 22.4 (SD = 3.7) for participants who are 18–29 years to 19.1 (SD = 2.5) for participants 50 years or older. We also observed a similar trend for participants with 12 years or more of education (Table 2).

For the main model with MoCA overall test score as the dependent variable (Model 1, Table 3), male gender was significantly associated with higher overall test score ($\hat{\beta }$ = 0.815, *p *= 0.015). Age was also significantly associated with overall test score ($\hat{\beta }$ = −0.048, *p* = 0.004). Education categories of 12, 13–15, and 16 years plus were all significantly associated with higher overall test scores relative to less than 12 years of education.

**TABLE 3:** Linear regression models for deriving age, education, and gender adjusted norms.

Model (#)	Test/Domain	Constant	Male gender	Age (years)	Education (12 years)	Education (13–15 years)	Education (16+ years)	$R^{2}$	RMSE
**1**	**MoCA**	$\hat{\beta }$(23.000) (21.399, 24.602) *p =* 0.000	$\hat{\beta }$(0.815) (0.166, 1.464) *p =* 0.014	$\hat{\beta }$(−0.048) (−0.081, −0.015) *p =* 0.005	$\hat{\beta }$(1.654) (0.368, 2.939) *p =* 0.012	$\hat{\beta }$(2.223) (0.925, 3.520) *p =* 0.001	$\hat{\beta }$(2.451) (1.328, 3.576) *p =* 0.000	0.105	2.837
**2**	**Visuospatial**	$\hat{\beta }$(2.354) (1.800, 2.908) *p =* 0.000	$\hat{\beta }$(−0.033) (−0.276, 0.208) *p =* 0.784	$\hat{\beta }$(−0.013) (−0.025, −0.000) *p =* 0.038	$\hat{\beta }$(0.354) (−0.074, 0.783) *p =* 0.105	$\hat{\beta }$(0.615) (0.171, 1.002) *p =* 0.007	$\hat{\beta }$(0.634) (0.266, 1.001) *p =* 0.001	0.057	1.012
**3**	**Executive functions**	$\hat{\beta }$(2.648) (2.161, 3.135) *p =* 0.000	$\hat{\beta }$(0.155) (−0.061, 0.370) *p =* 0.160	$\hat{\beta }$(−0.005) (−0.015, 0.005) *p =* 0.325	$\hat{\beta }$(0.336) (−0.834, 0.756) *p =* 0.116	$\hat{\beta }$(0.633) (0.159, 1.107) *p =* 0.009	$\hat{\beta }$(1.010) (0.675, 1.345) *p =* 0.000	0.142	0.932
**4**	**Attention**	$\hat{\beta }$(4.158) (3.597, 4.719) *p =* 0.000	$\hat{\beta }$(0.442) (0.213, 0.672) *p =* 0.000	$\hat{\beta }$(−0.006) (−0.016, 0.005) *p =* 0.305	$\hat{\beta }$(0.733) (0.231, 1.235) *p =* 0.004	$\hat{\beta }$(0.919) (0.440, 1.398) *p =* 0.000	$\hat{\beta }$(1.049) (0.643, 1.456) *p =* 0.000	0.134	1.027
**5**	**Language**	$\hat{\beta }$(3.737) (3.185, 4.289) *p =* 0.000	$\hat{\beta }$(0.131) (−0.087, 0.349) *p =* 0.239	$\hat{\beta }$(0.003) (−0.007, 0.014) *p =* 0.571	$\hat{\beta }$(0.457) (0.034, 0.880) *p =* 0.034	$\hat{\beta }$(0.715) (0.287, 1.142) *p =* 0.001	$\hat{\beta }$(0.817) (0.470, 1.165) *p =* 0.000	0.082	0.968
**6**	**Memory**	$\hat{\beta }$(3.879) (3.110, 4.648) *p =* 0.000	$\hat{\beta }$(0.239) (−0.073, 0.551) *p =* 0.133	$\hat{\beta }$(−0.017) (−0.033, −0.000) *p =* 0.038	$\hat{\beta }$(0.120) (−0.500, 0.739) *p =* 0.705	$\hat{\beta }$(0.128) (−0.508, 0.763) *p =* 0.693	$\hat{\beta }$(0.145) (−0.387, 0.677) *p =* 0.592	0.019	1.378
**7**	**Orientation**	$\hat{\beta }$(5.795) (5.492, 6.097) *p =* 0.000	$\hat{\beta }$(−0.061) (−0.019, 0.063) *p =* 0.335	$\hat{\beta }$(−0.004) (−0.012, 0.003) *p =* 0.282	$\hat{\beta }$(0.003) (−0.223, 0.229) *p =* 0.978	$\hat{\beta }$(0.100) (−0.113, 0.314) *p =* 0.356	$\hat{\beta }$(0.033) (−0.144, 0.211) *p =* 0.714	0.012	0.542

*Note*: $\hat{\beta }$ is the coefficient from a linear regression model or the estimate of the association between each sociodemographic variable and MoCA test, domain, or subdomain scores. $R^{2}$ is the coefficient of determination that denotes the proportion of the variation in the dependent variable that is accounted for by the independent variables in each model. RMSE is the root mean squared error for each model that assesses the average difference between values predicted by a model and the actual values. The overall *p* values for the statistical significance of each of the regression models are as follows: ^1^Model 1 (*p *< 0.001); ^2^Model 2 (*p *= 0.001); ^3^Model 3 (*p *= 0.001); ^4^Model 4 (*p *< 0.001); Model^5^ (*p *< 0.001); Model^6^ (*p *< 0.001); Model^7^ (*p *= 0.323).

The visuospatial domain (Model 2, Table 3) was negatively associated with age ($\hat{\beta }$ = −0.013, *p* = 0.038), and only education categories greater than 12 years were significantly associated with higher visuospatial domain score relative to less than 12 years of education.

Executive function score (Model 3, Table 3) was significantly associated with 13–15 years of education and with 16 years or more of education relative to less than 12 years of education.

Higher attention score (Model 4, Table 3) was associated with male gender ($\hat{\beta }$ = 1.131, *p* < 0.001). Relative to less than 12 years of education, all other education categories were associated with higher attention scores (Model 4, Table 3).

Similarly, relative to less than 12 years of education, all other education categories were associated with higher language scores (Model 5, Table 3).

Delayed memory score (Model 6, Table 3) was only associated with older age ($\hat{\beta }$ = −0.017, *p *= 0.033). Orientation score (Model 7, Table 3) was not significantly associated with age, gender, or education. Therefore, these were the only two models that were not significant overall (Table 3).

The age, gender, and education‐adjusted cut‐off scores for the MoCA overall test and its six domain scores based on the 5th, 10th, 15th, and 20th percentile rankings in our sample are presented in Table 4. The 5th percentile rankings of participants in our sample (Table 4) on the overall test score and its domains (rounded to the nearest integer) adjusted for gender, age, or level of education were as follows: MoCA (22), visuospatial (2), executive (2.5), attention (4), language (4), and delayed memory (3).

**TABLE 4:** Raw and adjusted Montreal Cognitive Assessment (MoCA) total and subdomain scores and corresponding values below the 5th, 10th, 15th, and 20th percentiles.

Test/Domain/Subdomain	Score type	Score	Percentile (%)			
			5	10	15	20
**MoCA**	Raw	*z*‐Score	−1.72	−1.25	−0.93	−0.77
		Observed	18.00	19.50	20.50	21.00
	Adjusted	*z*‐Score	−1.92	−1.35	−1.12	−0.91
		Expected	21.85	22.25	22.68	23.00
**Visuospatial**	Raw	*z*‐Score	−1.81	−1.32	−1.32	−0.84
		Observed	0.50	1.00	1.00	1.50
	Adjusted	*z*‐Score	−2.12	−2.12	−2.12	−2.12
		Expected	1.72	1.91	2.05	2.10
**Executive**	Raw score	*z*‐Score	−2.27	−1.27	−1.27	−1.27
		Observed	1.00	2.00	2.00	2.00
	Adjusted score	*z*‐Score	−3.90	−3.90	−2.60	−2.32
		Expected	2.50	2.54	2.69	2.81
**Attention**	Raw score	*z*‐Score	−1.85	1.85	−0.94	−0.94
		Observed	3.00	3.00	4.00	4.00
	Adjusted score	*z*‐Score	−4.34	−4.34	−3.92	−2.64
		Expected	4.03	4.27	4.48	4.71
**Language**	Raw	*z*‐Score	−1.57	−1.57	−0.57	−0.57
		Observed	3.00	3.00	4.00	4.00
	Adjusted	*z*‐Score	−2.91	−2.91	−1.83	−1.77
		Expected	3.85	3.93	4.05	4.27
**Delayed memory**	Raw	*z*‐Score	−1.77	−1.05	−1.05	−0.33
		Observed	1.00	2.00	2.00	3.00
	Adjusted	*z*‐Score	−1.68	−1.17	−0.98	−0.62
		Expected	3.12	3.23	3.28	3.32
**Orientation**	Raw	*z*‐Score	−1.21	−1.21	−1.21	−1.21
		Observed	5.00	5.00	5.00	5.00
	Adjusted	*z*‐Score	−1.33	−1.31	−1.26	−1.16
		Expected	5.54	5.56	5.59	5.60

### 4.1 Hypothetical Scenario

We demonstrate the use of our demographically adjusted normative cut‐off overall score of 22 for the MoCA test to screen for MCI in clinical practice using the following hypothetical example. A 45‐year‐old Qatari patient with 12 years of education obtained a raw score of 21 on the MoCA test during a routine MCI screening assessment. Instead of relying on the raw MoCA score of 21, the clinician proceeds to calculate an expected MoCA cut‐off score (*x*) for this patient by looking up the corresponding raw mean score and SD of a typical individual in the age category of 30–49 years with 12 years of education (mean = 23.4, SD = 2.6 as per Table 2) and plugging these values along with the *z*‐score of the 5th percentile (*z* = −1.92 as per Table 4) into the following equation (derived from Equation 1), we arrive at *x* = 28.4:

(3)

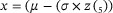




Given that 28.4 is above the mean population–derived adjusted cut‐off score of 22 (Table 4), this patient's population‐adjusted score is considered within the normal range. Notably, the clinician would have reached a different conclusion (i.e., this patient's score would be considered below the normal range) if he had compared the patient's raw score of 21 with the mean population‐derived adjusted cut‐off score of 22 from our study or against the recommended cut‐off score of 26 in the original MoCA study (Nasreddine et al. 2005).

## 5 Discussion

The present study set out to establish raw and sociodemographically adjusted population norms for the overall MoCA test and its domains in a representative sample of the healthy Arab adults residing in Qatar, which have not been established to date. Rounded to the nearest decimal, the raw mean and median overall MoCA test scores were 24. Furthermore, approximately 74% of the participants in our sample scored below the recommended cut‐off score of 26 for screening participants in English‐speaking samples (Nasreddine et al. 2005). On the basis of our norming procedure, we recommend using an overall test score of 22 (rounded to the nearest decimal) as the cut‐off for screening for MCI in healthy Arab adults irrespective of their gender, age, or level of education. This score represents the 5th percentile ranking of participants in our sample; that is, 5% of our sample scored 22 or lower on the MoCA, which is a relatively low score based on the adjusted score distribution of our healthy population sample.

A recent study reported normative data on the MoCA among community‐dwelling Saudi Arabians between 18 and 80 years of age (Muayqil et al. 2021). The mean scores on the original and basic (designed for illiterate people and those with lower educational achievements) versions of the test were similar to their score from the basic version; meanwhile, our adjusted cut‐off score based on the 5th percentile ranking was closer to the original score from the Saudi sample (Muayqil et al. 2021). In addition, our results were similar to findings from their multivariable regression analysis, showing a significant association between the MoCA overall score and age as well as years of education in both versions: MoCA test score improved with higher levels of education and worsened with increasing age (Muayqil et al. 2021).

In contrast to the Saudi study, which showed no gender differences in MoCA test scores among participants (Muayqil et al. 2021), males scored significantly higher on the MoCA overall test in our study, on average, compared to females, even after adjustment for age and education years. Overall, this finding was primarily driven by men performing better than women, mainly on tasks associated with attention. In contrast, gender did not significantly associate with performance on visuospatial/executive function or language domains. To date, the evidence in support of the role of gender in explaining cognitive screening test scores is mixed with some studies supporting gender differences (Engedal et al. 2021; Han et al. 2008; Measso et al. 1993; Mías et al. 2007; Ribeiro et al. 2010; Scazufca et al. 2009), whereas others have not (Bertolucci et al. 2001; Mathuranath et al. 2007; Thomann et al. 2018).

In our study, younger age and higher number of years of education were significantly associated with higher visuospatial scores, but only older age was significantly associated with worse delayed memory scores. A higher number (13 or more) of years of education was also significantly associated with higher executive function scores. No sociodemographic variables were associated with orientation domain scores in our sample, which is expected as the majority of participants in our sample were relatively healthy at the time of the interview. Additionally, previous studies indicated that impaired orientation was a stronger correlate of severe rather than mild cognitive dysfunction (Morris et al. 2001).

The mean language score in our study was lower than the mean language score reported in the original MoCA study (Nasreddine et al. 2005), which is consistent with other studies from the Arab region (Saudi Arabia and Lebanon) where participants’ language (including verbal fluency) domain scores were much lower on average than typically reported in English‐speaking countries (Hayek et al. 2020; Muayqil et al. 2021). Interestingly, only education was significantly associated with language domain score in our study, suggesting that scores on this domain will vary on the basis of the distribution of education level in the population.

Although the present findings were based on a relatively large and representative sample of Arab adults in the general population of Qatar, we had a small frequency of individuals who are 50+ years of age. Therefore, it is important that we acknowledge that because individuals in this age category are at higher risk of cognitive dysfunction, including MCI, their low numbers in our sample may introduce higher variability and thus lower precision in the corresponding estimate of the cognitive function norm for this group. Nevertheless, the norms provided by the present study will serve as a preliminary guide for health care providers dealing with diverse Arab populations in Qatar and beyond. Further validation against gold standard cognitive assessment tools in Arabic and against clinical samples and longitudinally is needed. For example, it would be important to know what percentage of those who screen positive for MCI on the Arabic MoCA test have a neurological disorder or are at risk to develop dementia or other brain disorders. Future research in Qatar and other Arab countries should also endeavor to identify MoCA domain scores and sociocultural factors that are prognostic for poor cognitive outcomes in initially healthy populations.

## 6 Conclusions

The present study is the first to report raw and sociodemographic‐adjusted norms for the overall MoCA test and six cognitive domains in a representative sample of healthy Arab adults residing in Qatar. We recommend the following sociodemographically adjusted 5th percentile cut‐off scores for MoCA overall test (22), visuospatial (2), executive (2.5), attention (4), language (4), and delayed memory (3), respectively. Scores below these 5th percentile cut‐offs may warrant further testing and clinical follow‐up for MCI.

## Author Contributions

**Iman Amro**: conceptualization, methodology, validation, writing–review and editing, writing–original draft. **Aisha M. Al Hamadi**: conceptualization, writing–original draft, methodology, data curation, writing–review and editing. **Alaa A. El Salem**: writing–review and editing, formal analysis. **Tawanda Chivese**: writing–original draft, formal analysis, writing–review and editing, methodology. **Stacy S. Wilkins**: conceptualization, supervision, data curation, writing–review and editing, writing–original draft, methodology. **Salma M. Khaled**: conceptualization, methodology, data curation, investigation, funding acquisition, writing–original draft, writing–review and editing, project administration, formal analysis; supervision.

## Conflicts of Interest

The authors declare no conflicts of interest.

### Peer Review

The peer review history for this article is available at https://publons.com/publon/10.1002/brb3.70287.

## Supporting information



Appendix 1. Programmed Arabic MoCA

Appendix 2. Sample Demographic Characteristics

## Data Availability

Data are available from the corresponding author (skhaled@qu.edu.qa) upon written request.
