# Supplementary material for: Population‐Based Norms for the Montreal Cognitive Assessment in Arab Adults
Source: Brain Behav. 2025 Feb 9;15(2):e70287. doi: 10.1002/brb3.70287 (PMC11807847; doi:10.1002/brb3.70287)
Supplement: Supplementary file 1 — Appendix 1. Programmed Arabic MoCA [file BRB3-15-e70287-s001.docx]

**Supplementary Materials:**

***Appendix 1*.** Programmed Arabic MoCA

# MoCA

**تقييم مونتريال المعرفي (MoCA)**

(MOCA_INTRO)

صُمم هذا الجزء من المقابلة لتقييم الوظائف المعرفية، وهو يقيّم الوظائف التالية: الانتباه، التركيز، الذاكرة، اللغة، الحساب والاهتداء. زمن الاختبار هو تقريباً عشر دقائق.

) مجري المقابلة: يجب تذكير المجيب بضرورة أجراء الفحص المعرفي في مكان هادئ بدون تواجد اشخاص أخرون لتجنب المقاطعة أثناء الفحص. (

(EDUCATIONYEARS)

قبل أن نبدئ بهذا الجزء من المقابلة، كم عدد سنوات التعليم التي حصلت عليها؟

(مجري المقابلة: أذا لم يتلقَ تعليماً ضع"0"، أذا أكمل المدرسة الابتدائية ضع"6"، أذا أكمل المرحلة الثانوية ضع"12")

____________________PROGRAMMER NUMBER OF YEARS ONLY

(VISUOSPATIAL1)

**) مجري المقابلة قم بالتأشير إلى المكان المناسب على الجهاز اللوحي واقرأ (:**

أريد منك أن ترسم خطاً بتناوب رقم وحرف مع احترام الترتيب الرقمي (التصاعدي) وترتيب الأبجدية. ابدأ من الرقم 1، وارسم الخط نحو حرف أ، ثم نحو رقم 2، واستمر بذلك. وانتهي هنا عند حرف ج.


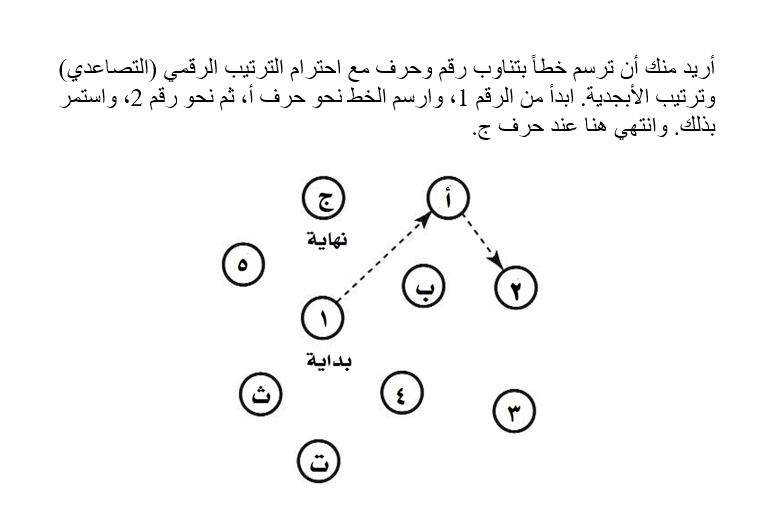
(VISUOSPATIAL2)

(كُتيب المقابلة، صفحة " أ "): أريد منك أن تنسخ هذا الرسم بأدق طريقة ممكنة.

(VISUOSPATIAL3)

(مجري المقابلة قم بالإشارة إلى المكان المحدد للرسم واقرأ (: أريد منك الآن أن ترسم ساعة حائط وتضع جميع الأرقام مع تعيين الساعة إلى 11 و10 دقائق.

(VISOUINSTRUCT)

(مجري المقابلة: لطفاً تأكد من أن المستجيب أكمل جميع الرسومات قبل الانتقال للسؤال التالي، أو عُد إلى السؤال غير المكتمل إذا كان هناك أي نقص)

1. استمر

(NAMING_INTRO)

(كُتيب المقابلة، صفحة "ب") هل يمكنك أن تذكر أسم الحيوانات التالية من اليمين إلى اليسار؟

(مجري المقابلة: سجل كل أسم ذكره المستجيب)

الصورة1 ________________(NAMING_1)

الصورة2 ________________(NAMING_2)

الصورة3 ________________(NAMING_3)

(MEMO1)

هذا اختبار للذاكرة. سوف أقرأ لك من الكلمات يجب أن تحفظها. انتبه جيداً وعندما أنتهي أريد منك أن تعيد أكبر عدد ممكن من هذه الكلمات التي يمكن أن تتذكرها، وبالتسلسل الذي تريده.

(مجري المقابلة: اقرأ الكلمات بمعدل كلمة كل ثانية، ضع علامة (X) عند كل كلمة أعادها المستجيب بشكل صحيح)

| لم يستطيع تذكر أي كلمة | أزرق | قرنفلة | مدرسة | مخمل | وجه |  |
| --- | --- | --- | --- | --- | --- | --- |
|  |  |  |  |  |  | اختبار 1 |

(MEMO2)

الآن سوف أقرأ لك قائمة الكلمات مرة أخرى. حاول أن تتذكر أكبر عدد ممكن منها بما فيها تلك التي ذكرتها في المرة الأولى."

(مجري المقابلة اقرأ الكلمات مرة أخرى ضع علامة (X)عند كل كلمة أعادها المستجيب بشكل صحيح.)

| لم يستطيع تذكر أي كلمة | أزرق | قرنفلة | مدرسة | مخمل | وجه | الكلمات |
| --- | --- | --- | --- | --- | --- | --- |
|  |  |  |  |  |  | اختبار2 |

[PROGRAMER: PLEASE ADD THE INSTRUCTIONS BELOW ON A SEPARATE PAGE]

(MEMOINTRUCT)

(مجري المقابلة قل: يجب أن تحفظ تلك الكلمات لأني سوف أطلب منك تذكرها في نهاية الاختبار)

اضغط 1. للاستمرار.

(ATTENTION1)

سوف أقرأ لك عدد من الأرقام وعندما أنتهي، أريد منك أن تعيد تلك الأرقام بنفس الترتيب الذي ذكرته.

(مجري المقابلة اقرأ الأرقام بمعدل رقم كل ثانية)

الأرقام هي: 2 – 1 – 8 – 5 – 4

[PROGRAMER: PLEASE OPTIONS SHOULD BE EMPTY SLOTS, ONLY NUMBERS ALLOWED, RANGED FROM 1 – 10]

|  | ATTENTION1_1 | ATTENTION1_2 | ATTENTION1_3 | ATTENTION1_4 | ATTENTION1_5 |
| --- | --- | --- | --- | --- | --- |
| سجل الرقم |  |  |  |  |  |

(ATTENTION2)

سوف أقرأ لك عدد من الأرقام وعندما أنتهي أريد منك أن تعيد تلك الأرقام بعكس الترتيب الذي ذكرته

(مجري المقابلة: اقرأ الأرقام بمعدل رقم كل ثانية)

الأرقام هي: 7 – 4 – 2

[PROGRAMER: PLEASE OPTIONS SHOULD BE EMPTY SLOTS, ONLY NUMBERS ALLOWED, RANGED FROM 1 – 10]

|  | ATTENTION2_1 | ATTENTION2_2 | ATTENTION2_3 |
| --- | --- | --- | --- |
| سجل الرقم |  |  |  |

(ATTENTION3)

سوف أقرأ لك من الأحرف، كلما أذكر حرف (أ) يجب أن تقرع (تطرق) بيدك مرة واحدة، وعندما أقول حرفاً غير حرف (أ) لا تقرع (تطرق) باليد.

(مجري المقابلة: اقرأ الأحرف بمعدل حرف كل ثانية، ضع علامة ✔عند الحرف الذي قرع عنده المستجيب)

[PROGRAMER: PLEASE REMOVE THE NUMBERS/CODES IN BLAISE]

| الاحرف | ف | ب | أ | س | م | ن | أ | ج | ك | ل | ب | أ | ف | أ | ك | د | ط | أ | أ | أ | ج | أ | م | و | ف | أ | أ | ب |
| --- | --- | --- | --- | --- | --- | --- | --- | --- | --- | --- | --- | --- | --- | --- | --- | --- | --- | --- | --- | --- | --- | --- | --- | --- | --- | --- | --- | --- |
| القرع عند حرف |  |  |  |  |  |  |  |  |  |  |  |  |  |  |  |  |  |  |  |  |  |  |  |  |  |  |  |  |

(ATTENTION4)

الآن أريد منك أن تبدأ بحساب 100 – 7 ثم تستمر بطرح 7 من كل جواب إلى أن أقول لك أن تتوقف.

(مجري المقابلة عند الضرورة اقرأ: أريد منك أن تبدأ بحساب 100 – 7 ثم تستمر بطرح 7 من كل جواب إلى أن أقول لك أن تتوقف، ضع علامة X عند الرقم إذا ذكره المستجيب)

[PROGRAMER: PLEASE OPTIONS SHOULD BE EMPTY SLOTS, ONLY NUMBERS ALLOWED, RANGED FROM 0 – 100]

|  | ATTENTION4_1 | ATTENTION4_2 | ATTENTION4_3 | ATTENTION4_4 | ATTENTION4_5 |
| --- | --- | --- | --- | --- | --- |
| الإجابة |  |  |  |  |  |

(LANGUAGE1)

سوف أقرأ لك الآن جملة وأريد منك أن تعيدها بعدي: "الفأر يختبئ دائماً تحت المقعد عندما يدخل القط الغرفة."

(مجري المقابلة: يجب أن تكون الإعادة صحيحة تَمامًا بدون إِنقاص شيء أو تبديل كلمة بكلمة أو إضافة شيء الى الجملة)

1- كرر الجملة بشكل صحيح

5- لم يكررها بشكل صحيح

(LANOUAGE2)

الآن سوف أقرأ جملة ثانية ويجب أن تعيدها أيضاً: "أبو حمد زار جاره واطمأن عن صحته."

(مجري المقابلة: يجب أن تكون الإعادة صحيحة تَمامًا بدون إِنقاص شيء أو تبديل كلمة بكلمة أو إضافة شيء الى الجملة)

1- كرر الجملة بشكل صحيح

5- لم يكررها بشكل صحيح

(LANOUAGE3)

اذكر لي أكبر عدد ممكن من كلمات التي تبدأ بحرف ال (ف) خلال دقيقة، ما عدا الأسماء والأرقام واعراب الأفعال مثل: (أكل، أكلنا، أكلوا) والكلمات التي تنتمي لنفس العائلة مثل (عظيم – عظمة- عظماء).

(مجري المقابلة: سجل كل الكلمات التي ذكرها المستجيب)

____________

[PROGRAMER: FOR **ABSTRACTION1**, **ABSTRACTION2** AND **ABSTRACTION3** IF ANY OF THEM = 2, SHOW **ABSTRACTION** ONCE ONLY]

(ABSTRACTION1)

ما هو وجه الشبه بين البرتقال والموز

1. فواكه، فاكهه، fruits.
2. أخرى. حدد _________

(مجري المقابلة: لا تعطي أي تعليمات أو دلائل إضافية)

(ABSTRACTION2)

الآن قل لي ما هو وجه الشبه بين القطار والدراجة

1. مواصلات، تنقل، وسيلة مواصلات، ...
2. أخرى، حدد _____________

(مجري المقابلة: لا تعطي أي تعليمات أو دلائل إضافية)

(ABSTRACTION3)

الآن قل لي ما هو وجه الشبه بين المسطرة والساعة

1. أداة للقياس، قياس، مقاييس..
2. أخرى، حدد ____________

(مجري المقابلة: لا تعطي أي تعليمات أو دلائل إضافية)

(ABSTRACTION1a)

هل يمكنك اعطائي وجه تشابه آخر بين البرتقال والموز؟

سجل الإجابة: ______________

(ABSTRACTION2a)

هل يمكنك اعطائي وجه تشابه آخر بين الدراجة والقطار؟

سجل الإجابة: ______________

(ABSTRACTION3a)

هل يمكنك اعطائي وجه تشابه آخر بين المسطرة والساعة؟

سجل الإجابة: ______________

لقد قرأت لك سلسة من الكلمات من قبل وطلب

ت منك ان تتذكرها. أعد لي الآن جميع الكلمات التي تتذكرها؟ (MEMO3_1)

(مجري المقابلة ضع علامة ✔عند الكلمات التي ذكرت بشكل صحيح)

| أزرق  MEMO3_2e | قرنفلة  MEMO3_2d | مدرسة  MEMO3_2c | مخمل  MEMO3_2b | وجه MEMO3_2a | الكلمات |
| --- | --- | --- | --- | --- | --- |
|  |  |  |  |  | الكلمات التي ذكرت |

PROGRAMMER: FOR ANY MISSING IN THE ABOVE, CORRESPONDING PROMPTS MEMO3_2aa-MEMO3_2ee SHOWN BELOW SHOULD POP UP

(MEMO3_2)

(مجري المقابلة: إذا لم يتذكر المستجيب أي من الكلمات يمكنك أن تساعده بالدلائل اولاً)

سأعطيك دليل يساعدك على التذكر، الكلمة تتعلق ب...

|  | الدليل | الكلمات التي تذكرها المجيب |
| --- | --- | --- |
| MEMO3_2aa | جزء من الجسم |  |
| MEMO3_2bb | قماش |  |
| MEMO3_2cc | مبنى |  |
| MEMO3_2dd | زهرة |  |
| MEMO3_2ee | لون |  |

PROGRAMMER: FOR ANY MISSING IN THE ABOVE, CORRESPONDING PROMPTS MEMO3_2aaa-MEMO3_2eee SHOWN BELOW SHOULD POP UP

(MEMO3_3)

(مجري المقابلة: إذا لم يتذكر المستجيب أي من الكلمات يمكنك أن تقرأ له الخيارات) سأعطيك دليل يساعدك على التذكر،

هل كانت الكلمة:

|  | الخيارات | الكلمات التي تذكرها المجيب |
| --- | --- | --- |
| MEMO3_2aaa | الأنف، الوجه، اليد |  |
| MEMO3_2bbb | قطن، مخمل، حرير |  |
| MEMO3_2ccc | مدرسة، مصنع مطعم |  |
| MEMO3_2ddd | قرنفلة، زئبقة، ياسمين |  |
| MEMO3_2eee | أحمر، أزرق، أخضر |  |

(ORIENTATION1)

[PROGRAMMER: PLEASE DISPLAY ON THE SAME SCREEN]

قل لي ما هو تاريخ اليوم؟

(مجري المقابلة، إذا كان الجواب غير كافي اقرأ: قل لي السنة والشهر واليوم الصحيح)

[PROGRAMER: PLEASE DROP DOWN LIST FOR ALL SLOTS]

السنة________

الشهر____________

1. 1 / January / يناير / كانون الثاني
2. 2 / February / فبراير / شباط
3. 3 / March / مارس / آذار
4. 4 / April / ابريل / نيسان
5. 5 / May/ مايو / أيار
6. 6 / June / يونيو / حزيران
7. 7 / July / يوليو / تموز
8. 8 / August / أغسطس / آب
9. 9 / September/ سبتمبر / أيلول
10. 10 / October / أكتوبر / تشرين الأول
11. 11 / November / نوفمبر / تشرين الثاني
12. 12 / December / ديسمبر / كانون الأول

تاريخ اليوم______ [RANGE FROM: 1 – 31]

أي يوم فالأسبوع؟

**(مجري المقابلة: لا تقرأ الخيارات)**

1. الأحد
2. الاثنين
3. الثلاثاء
4. الأربعاء
5. الخميس
6. الجمعة
7. السبت

ORIENTATION2_a)

الآن قل لي ما هو اسم المكان الذي نحن فيه حالياً؟ (مثلاً: مستشفى، عيادة، مكتب)

اسم المكان__________

(ORIENTATION2_b)

وفي أي مدينة يقع؟

المدينة______________
