# Supplementary material for: Population‐Based Norms for the Montreal Cognitive Assessment in Arab Adults
Source: Brain Behav. 2025 Feb 9;15(2):e70287. doi: 10.1002/brb3.70287 (PMC11807847; doi:10.1002/brb3.70287)
Supplement: Supplementary file 2 — Appendix 2. Sample Demographic Characteristics [file BRB3-15-e70287-s002.docx]

***Supplementary Materials:***

***Appendix 2***. Sample Demographic Characteristics

| **Variable** | **Categories** | **Frequency**  **(n)** | **Percentage**  **(%)** |
| --- | --- | --- | --- |
| **Gender** | Male | 151 | 49.1 |
|  | Female | 157 | 50.9 |
|  | Missing | 0 | 0.0 |
| **Household type** | Qatari | 47 | 15.3 |
|  | Non-Qatari | 261 | 84.7 |
|  | Missing | 0 | 0.0 |
| **Age**  **(Years)** | 18-29 | 62 | 20.1 |
|  | 30-49 | 198 | 64.3 |
|  | 50+ | 48 | 15.6 |
|  | Missing | 0 | 0.0 |
| **Education** | Secondary/Diploma | 123 | 39.9 |
|  | Graduate | 150 | 48.7 |
|  | Bachelor | 34 | 11.1 |
|  | Missing | 1 | 0.3 |
| **Marital status** | Never married | 40 | 13.0 |
|  | Ever married | 268 | 87.0 |
|  | Missing | 0 | 0.0 |
| **Income**  **(Qatari Riyals)** | Less than 20K | 208 | 67.5 |
|  | 20 K + | 87 | 28.3 |
|  | Missing | 13 | 4.2 |
